# Differential regulation of apical–basolateral dendrite outgrowth by activity in hippocampal neurons

**DOI:** 10.3389/fncel.2015.00314

**Published:** 2015-08-11

**Authors:** Yang Yuan, Eunju Seong, Li Yuan, Dipika Singh, Jyothi Arikkath

**Affiliations:** ^1^Developmental Neuroscience, Munroe-Meyer Institute, University of Nebraska Medical Center, Omaha, NEUSA; ^2^Department of Pharmacology and Experimental Neuroscience, University of Nebraska Medical Center, Omaha, NEUSA

**Keywords:** dendrite, apical–basolateral, pyramidal neuron, neuronal plasticity, synapse development

## Abstract

Hippocampal pyramidal neurons have characteristic dendrite asymmetry, characterized by structurally and functionally distinct apical and basolateral dendrites. The ability of the neuron to generate and maintain dendrite asymmetry is vital, since synaptic inputs received are critically dependent on dendrite architecture. Little is known about the role of neuronal activity in guiding maintenance of dendrite asymmetry. Our data indicate that dendrite asymmetry is established and maintained early during development. Further, our results indicate that cell intrinsic and global alterations of neuronal activity have differential effects on net extension of apical and basolateral dendrites. Thus, apical and basolateral dendrite extension may be independently regulated by cell intrinsic and network neuronal activity during development, suggesting that individual dendrites may have autonomous control over net extension. We propose that regulated individual dendrite extension in response to cell intrinsic and neuronal network activity may allow temporal control of synapse specificity in the developing hippocampus.

## Introduction

Pyramidal neurons in the hippocampus and cortex have a typical morphology that includes multiple dendrites and, in most cases, single axons. Pyramidal neurons have a characteristic appearance that includes a pyramidal-shaped cell body from which a major primary dendrite, the apical dendrite, extends and gradually tapers away from the soma to terminate in a branched pattern known as an apical tuft (Spruston, 2008). Several minor dendrites, the basal dendrites, arise from the base of the pyramidal soma. Thus, these neurons have an intrinsic dendritic polarity, in addition to axon-dendrite polarity. Dendrites represent the major sources of information input into neurons via their ability to host synapses. Apical and basolateral dendrites differ in size, electrical conductivity and receptor and channel distribution (Horton and Ehlers, 2003). The asymmetry of the dendritic architecture is intimately linked to the processing capabilities of the neurons (Branco and Hausser, 2011; Bittner et al., 2012; Xu et al., 2012; Menon et al., 2013). The ability of neurons to generate and maintain dendrite polarity is critical, since the polarity of the dendrite dictates synaptic input specificity. For example, the hippocampal CA3 pyramidal neurons receive mossy fiber synapses onto their proximal apical dendrite and excitatory synapses from stellate cells of layer II of the entorhinal cortex onto their distal apical dendrite. Other CA3 axon collaterals synapse onto the remainder of the apical and the entire basal dendrite (Williams et al., 2011). Unlike the molecular mechanisms that govern axon-dendrite polarity (de Anda et al., 2005), much less is known about the cellular control of dendrite polarity leading to the generation and maintenance of the apical and basolateral dendrites in these neurons and dendrite compartmentalization.

It is likely that both activity and cell intrinsic factors cooperate to sculpt the developing dendritic arbor to generate and maintain its asymmetry. While some cell intrinsic factors that regulate dendrite polarity have been identified, the influence of neuronal activity on the asymmetry of the developing dendritic arbor remains obscure. Additionally, it remains unclear if all dendrites are similarly influenced by activity or dendrites have an asymmetric response to neuronal activity.

We examined the establishment of dendrite polarity and the influence of altering cell intrinsic and network activity on differential extension of apical and basolateral dendrites in a cultured primary neuron model during development. This is a widely used cell culture model and studies indicate that these neurons provide a good model system for these studies since dendrite asymmetry is maintained *in vitro* (Horton et al., 2005; Tran et al., 2009; Wu et al., 2015). In this model, apical and basolateral dendrites are defined by length. Our results indicate that while dendrite asymmetry is established early in development and maintained during growth, apical and basolateral dendrites in developing pyramidal neurons have differential net extension in response to cell intrinsic and neuronal network activity.

Our data are consistent with a model in which individual dendrites may have differential responses to cell intrinsic and network neuronal activity. Further, individual dendrites may have autonomous control over extension. This may provide an additional mechanism for temporal control of synapse specificity in the developing hippocampus.

## Materials and Methods

### Rat Hippocampal Primary Culture

Rat hippocampal primary cultures were prepared from E18–19 rats as previously described (Beaudoin et al., 2012). Neurons were maintained in serum-free media containing B-27 supplement (Invitrogen). All animal manipulations were performed in compliance with the UNMC approved protocols.

### Transient Transfection

Neurons were transfected with Lipofectamine 2000 (Invitrogen) as previously described (Arikkath et al., 2009; Beaudoin et al., 2012). The Kir2.1 and mutant Kir2.1 plasmids were a kind gift from Dr. Venki Murthy (Harvard University). For the Kir studies described in **Figure 3**, the neurons were transfected with the plasmids on DIV 0 or 1 and fixed on DIV 7 or transfected on DIV 7 and fixed on DIV 14. Neurons were also immunostained with anti-αCaMK2 antibody to identify glutamatergic neurons for quantitation (**Figures 3–5**). For data in **Figures 3F, 4F**, and **5F** neurons were identified by morphology.

### Antibodies

MAP2 – Millipore MAB3418, anti-αCaMK2 – Millipore 05-532, GM130- BD Transduction lab 610822, GABA – Sigma–Aldrich A 2052. Ctip2 and Prox1 – (Williams et al., 2011).

### Chemical Treatment of Neurons

Primary neurons in culture were treated with 50 mM KCl for 24 h or 1 m TTX (tetrodotoxin) (Tocris) for 5 days prior to fixation (DIV 7) and microscopy. For the data shown in **Figure 4F**, neurons were treated for 24 h with TTX.

### Image Acquisition and Preparation

After fixation, neurons were mounted in ProLong Gold antifade reagent (Invitrogen). Imaging was performed on an inverted Zeiss LSM700 microscope with 10X and 20X objectives as previously described (Yuan et al., 2013). LSM files obtained from the imaging software were converted into TIFF files and put together in Adobe Photoshop with minimum image manipulation. When image manipulation was needed, only to adjust intensity, the adjustment was applied to the entire image and not to parts of the image.

### Quantitation of Dendritic Arbors

Dendrite arbors were quantitated using ImageJ with or without the NeuronJ plugin, as previously described (Arikkath et al., 2008). The longest and second longest dendrites were visually identified before quantitation. The length of dendrite described in the figures indicates the total length of the dendrite including the higher order branches, except in **Figure 2**. For the data shown in **Figure 2**, only the longest shafts of the primary or secondary dendrites were analyzed. Note that at this stage of development, the primary shaft length is quite comparable to the entire dendrite length, since there is limited branching of the primary dendrite. For data in **Figure 2**, data was obtained from 287 neurons from three independent experiments. All data were obtained from three or more independent experiments and a total of 21–25 neurons for total dendritic length in **Figures 3A–E** and 23 to 45 neurons (**Figures 4A–E** and **5A–E**), for each condition, for all other experiments described. For **Figures 3F, 4F**, and **5F**, data was obtained from 36 to 44 neurons for each condition from three or four independent experiments.

### Statistical Analysis

Statistical analysis was performed using GraphPad Prism or Excel. The one-way ANOVA analysis in **Figures 3, 4F**, and **5F** was performed using the Greenhouse–Geisser correction and Dunnet’s multiple comparison test. *P* < 0.05 was considered significant. Student’s *t*-test was performed using two-tailed *t*-test assuming unequal variances.

## Results

**FIGURE 1:**
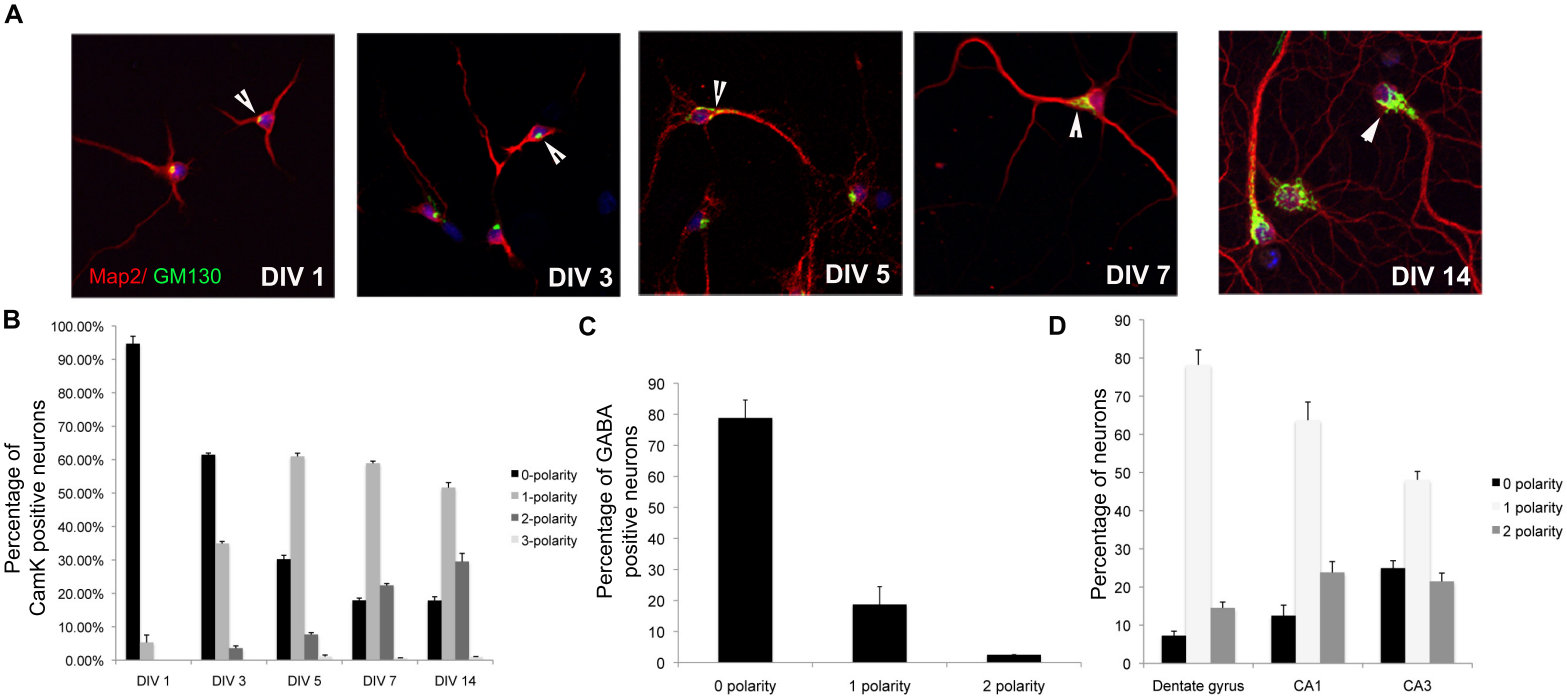
**Temporal stabilization of Golgi polarity relative to dendrite polarity. (A)** Representative images of primary neurons in culture immunostained with antibodies to GM130 and MAP2 at days *in vitro* indicated. Arrowheads indicate Golgi polarization. **(B)** Percentage of αCamK2 positive neurons with 0, 1, or 2 Gm130 polarities. **(C)** Percentage of GABAergic positive neurons with 0, 1, or 2 Gm130 polarities. **(D)** Percentage of CA1/CA2/DG neurons with 0, 1, or 2 Gm130 polarities (Error bars represent SD).

Pyramidal neurons in the hippocampus have distinct apical and basolateral dendrites. This asymmetry is recapitulates *in vitro* and apical and basolateral dendrites are distinguished by their lengths, with the apical being the longest and the basolateral being the second longest (Miao et al., 2013). It has previously been demonstrated that the Golgi apparatus is polarized toward the apical dendrite *in vitro*. We took advantage of this to examine the establishment of apical–basolateral dendrite polarity *in vitro* (Horton et al., 2005, 2006). Rat primary neurons *in vitro* were immunostained with antibodies to GM130, a Golgi marker, and the polarization of the Golgi relative to the longest dendrite was examined at different days *in vitro*. Neurons were also co-stained with antibodies to αCaMK2 to identify excitatory neurons and MAP2, a dendrite marker. In young neurons (DIV 1), the majority of neurons had Golgi apparatus that was not polarized toward a single dendrite (0 polarity) (**Figure 1A**). As development proceeded, the number of neurons with 0 polarity decreased with a concomitant increase in neurons with the Golgi polarized toward a single dendrite. The number of neurons with the Golgi polarized toward a single dendrite was not significantly different between DIV 5 and DIV 7. A small percentage of neurons demonstrated Golgi polarization toward two dendrites. This proportion increased with neuronal maturity. These results suggest that the temporal stabilization of the Golgi apparatus polarity relative to dendrite polarity occurs at around DIV 5 in these cultures (**Figure 1B**). Interestingly, unlike glutamatergic neurons, the GABAergic neurons did not have a polarized Golgi apparatus, consistent with the lack of apical–basolateral dendrite polarity in these neurons (**Figure 1C**) (Horton et al., 2005). We also took advantage of differential labeling of CA1/CA3/DG neurons by Prox1 and Ctip2 (Williams et al., 2011) to identify the subtype of neuron and examined Golgi polarity (**Figure 1D**). In all the three types of neurons, the Golgi was predominantly polarized toward a single dendrite.

**FIGURE 2:**
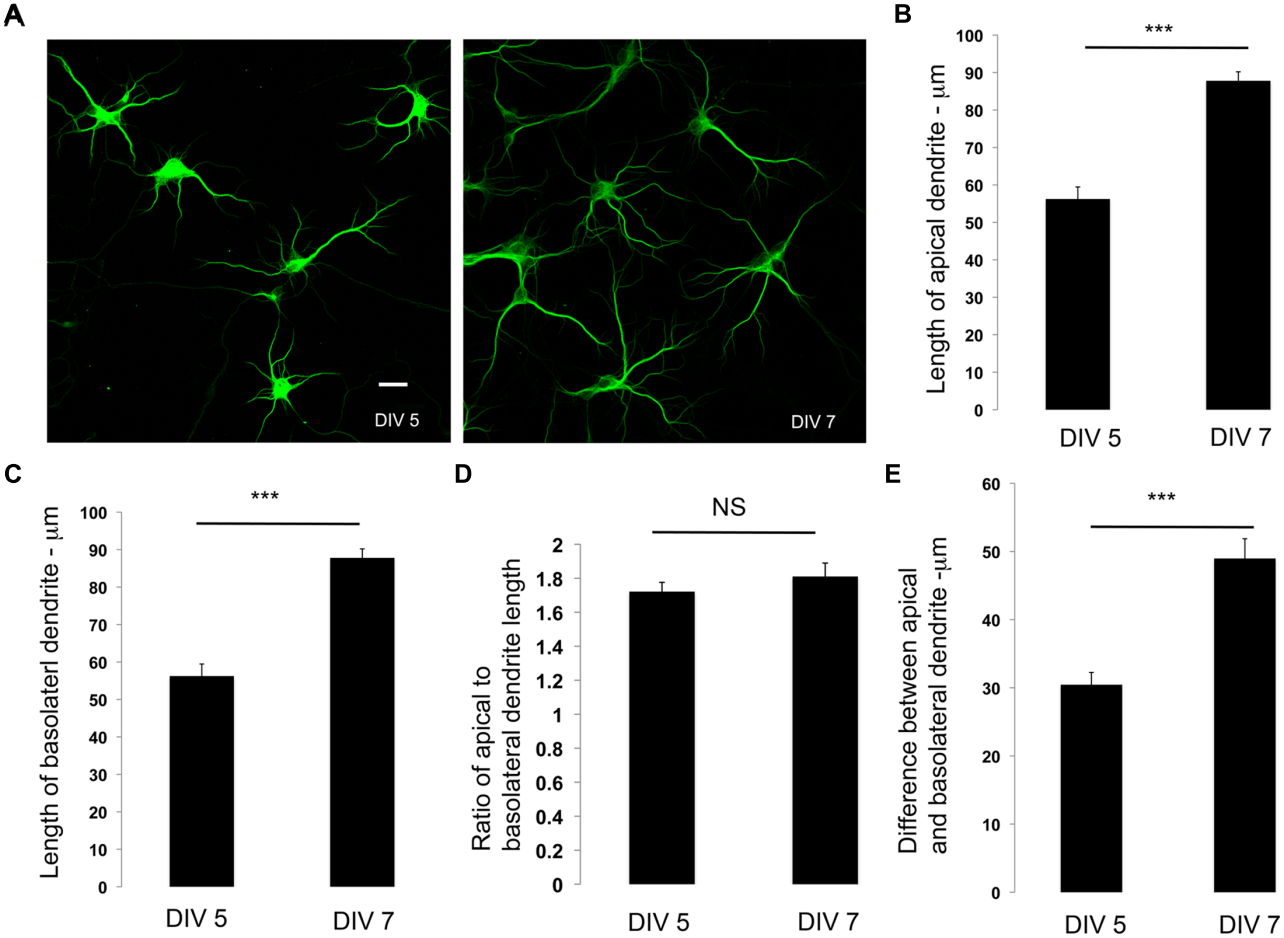
**Dendrite polarity is maintained during development. (A)** Representative images of primary neurons in culture immunostained with antibodies to MAP2 at days *in vitro* indicated. **(B)** Lengths of apical dendrites at DIV 5 and 7. **(C)** Length of basolateral dendrites at DIV 5 and 7. **(D)** Differences in apical and basolateral dendrites at DIV 5, and 7. **(E)** Ratio of primary to secondary dendrites at DIV 5 and 7 (Error bars represent SEM, Student’s *t*-test, *P* < 0.0005 -***, scale bar 20 microns.

Our data suggest that dendrite polarity is apparent and established at DIV 5. We examined if dendrite polarity is maintained during development. We examined the lengths, the ratio between the lengths and the differences between the lengths of apical and basolateral dendrites in developing neurons at DIV 5 and DIV 7. Primary neurons in culture were immunostained with antibodies to MAP2, examined by confocal microscopy (**Figure 2A**) and lengths quantitated. The apical and basolateral dendrites were visually identified by length. As expected, the lengths of both the apical and basolateral dendrites increased over time (**Figures 2B,C**). Interestingly, the ratio between the apical and basolateral dendrites was not significantly altered over time (**Figure 2D**), although the difference between the apical and basolateral dendrites increased with development (**Figure 2E**). These results suggest that, once the polarization of the dendrites is established, it is maintained during development. There is, however, one possible caveat for this interpretation. Developing dendrites are actively extending and retracting before they achieve their final dendritic arbor. In these studies, the definition of apical and basolateral is based on the length of individual dendrites. However, it is possible that during development, there may be a switch in the lengths of individual dendrites, such that the longest and second longest dendrites at early time points may switch to the second longest and longest, respectively over time. However, regardless of whether this switching does or does not occur, the ratio between the apical and basolateral dendrites is maintained, suggesting that the developing dendritic arbor has active mechanisms that maintain dendrite asymmetry.

**FIGURE 3:**
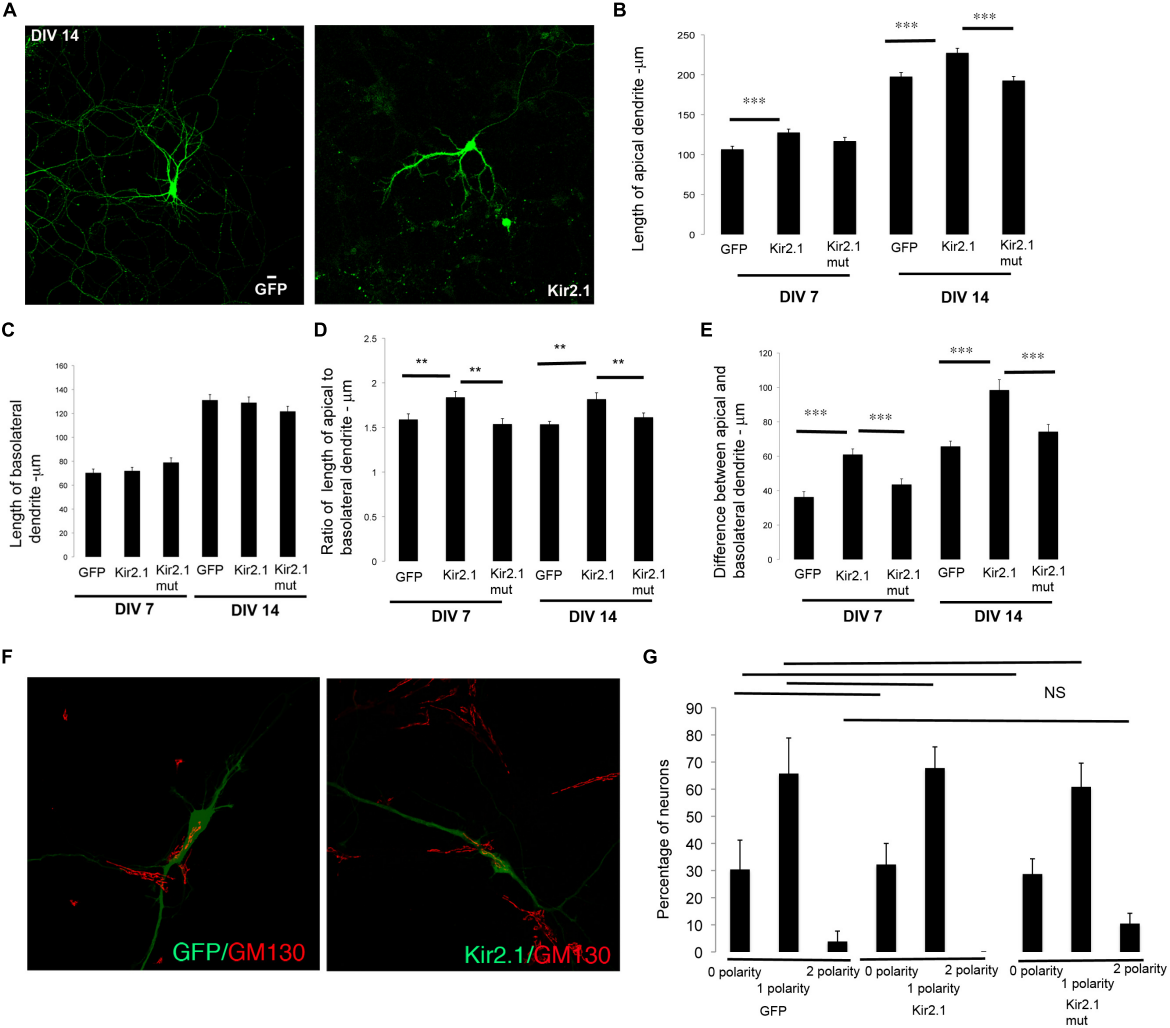
**Inhibition of cell intrinsic neuronal activity chronically selectively promotes net extension of apical dendrites. (A)** Representative confocal images of neurons transfected with GFP or Kir2.1 (DIV 7). **(B)** Length of apical dendrite. **(C)** Length of basolateral dendrite. **(D)** Ratio of lengths of apical to basolateral dendrites, and **(E)** Difference between apical and basolateral dendrite lengths in neurons expressing GFP, Kir2.1 or mutant form of Kir2.1 fixed at DIV 7 or DIV 14. **(F)** Confocal images of neurons expressing GFP or GFP and Kir2.1 and immunostained with anti-GM130. **(G)** Percentage of neurons with Golgi polarized toward 0, 1, or 2 dendrites in neurons expressing GFP or Kir2.1 or mutant form of Kir2.1, fixed at DIV 7. (Error bars represent SEM, One way Anova, *P* < 0.005 -**, *P* < 0.0005 -***, scale bar – 20 microns).

To examine the effects of inhibiting cell intrinsic neuronal activity, we expressed the inward-rectifier potassium channel, Kir2.1 (Hartman et al., 2006) in developing neurons. Developing neurons in culture were transfected with a plasmid encoding GFP, Kir2.1 or a mutant (non-conducting) form of Kir2.1 at DIV 0–1 and fixed at DIV 7. The neurons were subjected to imaging and lengths of apical and basolateral dendrites were obtained. In neurons expressing Kir2.1, the total length of the apical dendrite was significantly increased in comparison to control, while the length of the basolateral dendrite was unaltered (**Figures 3A–C**). These differences were significant enough to affect the ratio of the lengths of the apical to basolateral dendrites (**Figure 3D**). In addition, the differences in lengths between the apical and basolateral dendrites were significantly increased with expression of Kir2.1 (**Figure 3E**). This is likely reflecting the enhancement of the apical dendrite. In neurons expressing the Kir2.1 mutant that is non-conducting, the length of the apical dendrite was not significantly different from the control neurons. Similar results were obtained in older neurons (transfected at DIV 7 and fixed at DIV 14) expressing these constructs (**Figures 3B–E**). Interestingly, the polarization of the Golgi toward the dendrite was not significantly affected by expression of the Kir2.1, since the number of neurons with zero, one, or two Golgi polarities was not significantly altered in comparison to the GFP transfected neurons (**Figures 3F,G**). Taken together, these results indicate that inhibition of cell intrinsic neuronal activity selectively enhances the net extension of the apical dendrite without significantly affecting the extension of the basolateral dendrites in both developing and mature hippocampal neurons.

**FIGURE 4:**
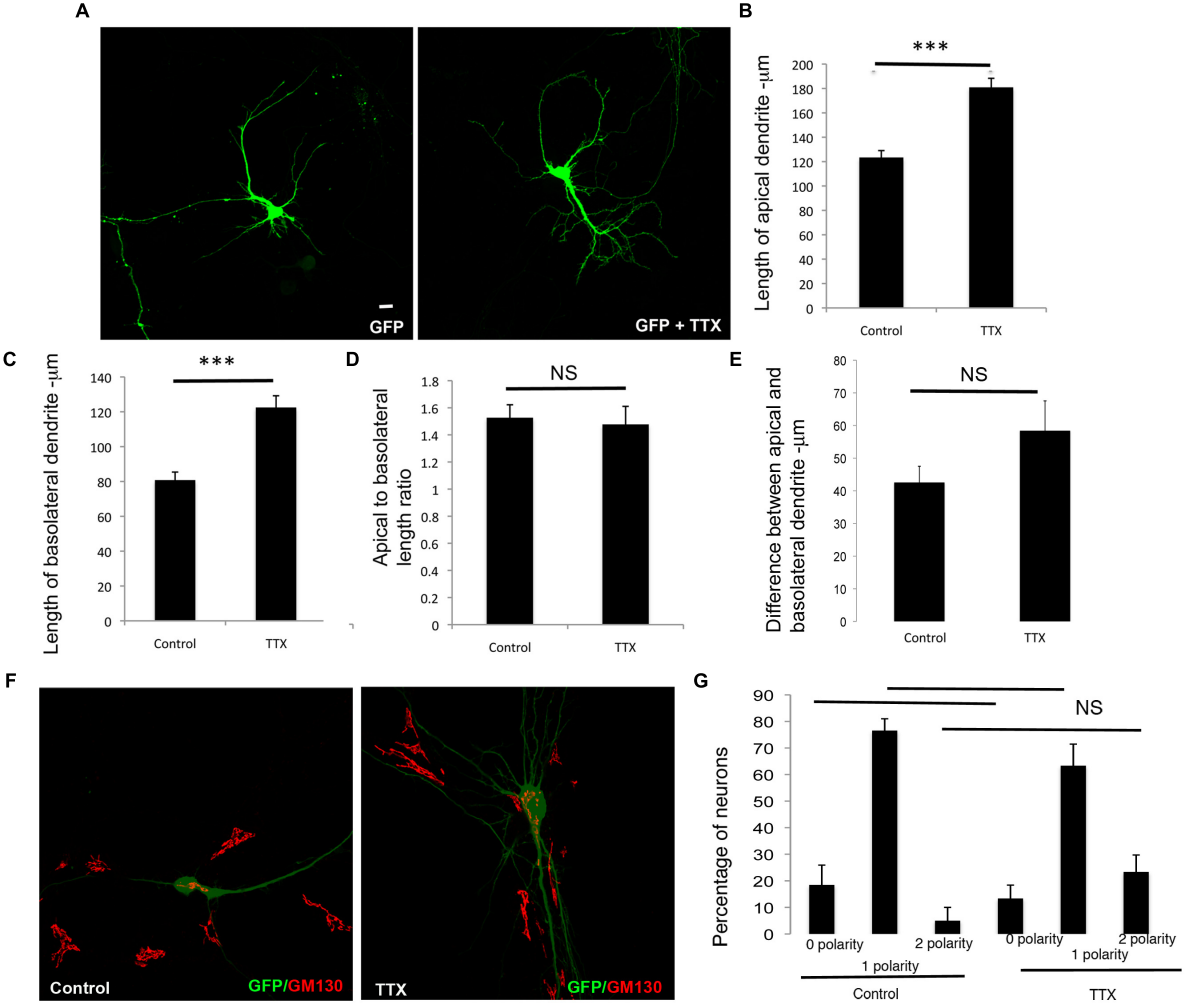
**Global chronic inhibition of activity promotes net extension of both apical and basolateral dendrites. (A)** Representative confocal images of neurons transfected with GFP and chronically treated with or without TTX. **(B)** Length of apical dendrite. **(C)** Length of basolateral dendrite. **(D)** Ratio of lengths of apical to basolateral dendrites, and **(E)** Difference between apical and basolateral dendrite lengths in neurons expressing GFP chronically treated without or with TTX at DIV 7. (Error bars represent SEM, Student’s *t*-test, *P* < 0.0005 -***, scale bar – 20 microns). **(F)** Confocal images of neurons expressing GFP treated without or with TTX and immunostained with anti-GM130. **(G)** Percentage of neurons with Golgi polarized toward 0, 1, or 2 dendrites in control or TTX treated neurons expressing GFP. (Error bars represent SEM, One way ANOVA, *P* < 0.0005 -***, scale bar – 20 microns).

We similarly examined the effects of inhibition of global network activity by TTX treatment on the differential extension of dendrites. In striking contrast to the effects observed with cell intrinsic inhibition of neuronal activity, TTX treatment resulted in an increase in the net extension of both apical and basolateral dendrites (**Figures 4A–C**). However, this did not significantly alter the ratio of the apical to basolateral dendrite lengths (**Figure 4D**). In addition, the differences between the apical and basolateral dendrite lengths were not significantly altered (**Figure 4E**), although there appeared to be a trend toward increase with TTX. Similar to the Kir2.1 expression, the polarization of the Golgi toward the dendrite was not significantly affected by TTX treatment, since the number of neurons with zero, one or two Golgi polarities was not significantly altered in comparison to the untreated neurons (**Figures 4F,G**). Thus, global inhibition of neuronal activity promotes the net extension of both apical and basolateral dendrites. Taken together with the results in **Figure 3**, these results demonstrate that the net extension of apical and basolateral dendrites are differentially affected by inhibition of cell intrinsic versus neuronal network activity.

**FIGURE 5:**
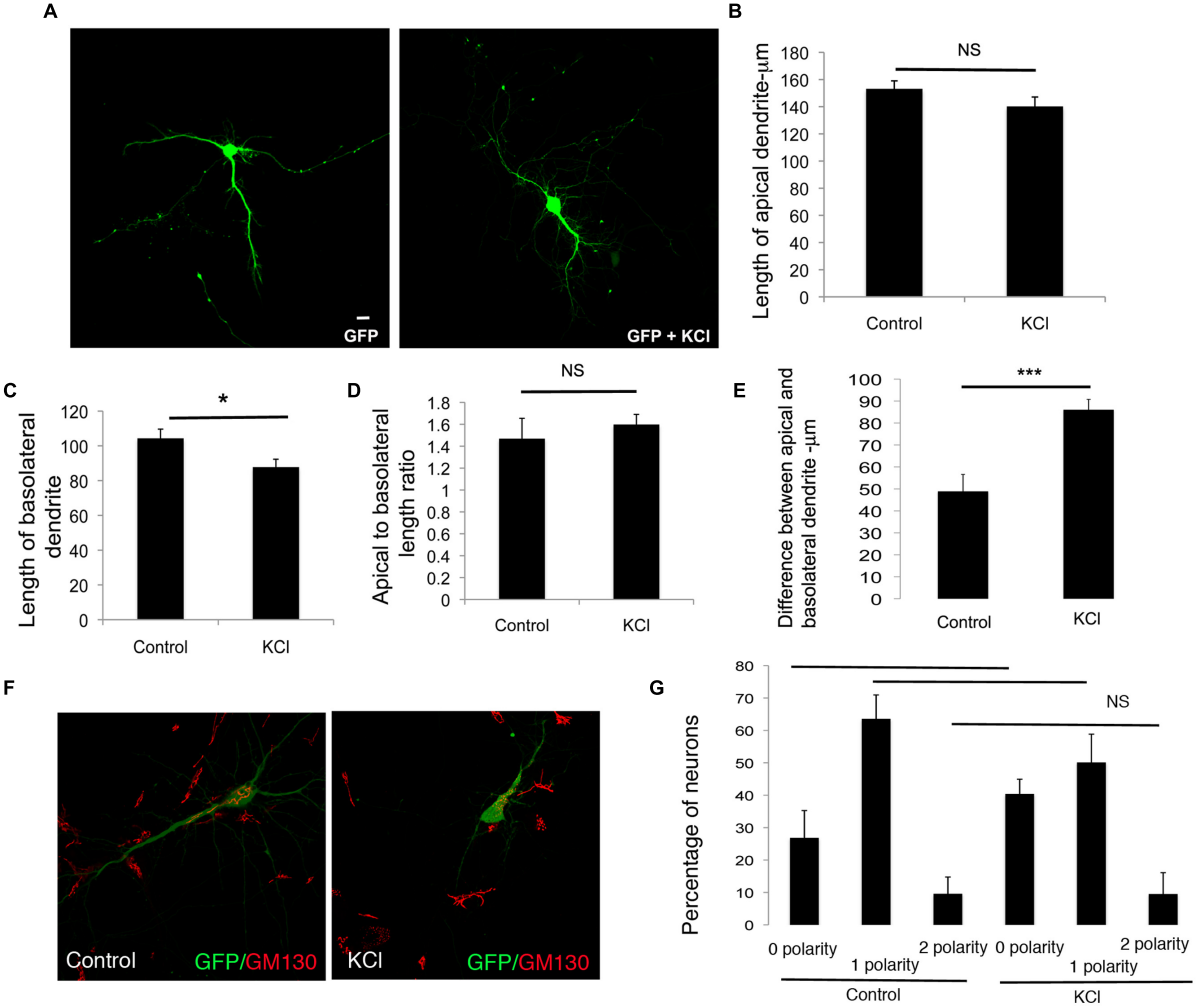
**Enhancing global neuronal activity suppresses net extension of basolateral dendrite extension in developing neurons. (A)** Representative confocal images of neurons transfected with GFP and treated with or without KCl. **(B)** Length of apical dendrite. **(C)** Length of basolateral dendrite. **(D)** Ratio of lengths of apical to basolateral dendrites, and **(E)** Total dendritic length in neurons expressing GFP chronically treated without or with KCl at DIV 7. (Error bars represent SEM, Student’s *t*-test, *P* < 0.05, <0.005 -**, *P* < 0.005 -***, scale bar – 20 mm). **(F)** Confocal images of neurons expressing GFP treated without or with KCl and immunostained with anti-GM130. **(G)** Percentage of neurons with Golgi polarized toward 0, 1, or 2 dendrites in control or KCl treated neurons expressing GFP. (Error bars represent SEM, One way ANOVA, *P* < 0.05 -*, *P* < 0.0005 -***, scale bar – 20 microns).

We similarly examined the effects of enhancement of global neuronal activity by KCl treatment on differential dendrite extension. In neurons treated with KCl, the net extension of the apical dendrite showed a trend toward reduction, however, these differences were not significant. In contrast, the length of the basolateral dendrite was significantly reduced (**Figures 5A–C**). These changes did not significantly affect the apical to basolateral dendrite length ratio (**Figure 5D**). However, there was a significant increase in the difference in length of the apical and basolateral dendrites with KCl treatment (**Figure 5E**). Although the Golgi apparatus was partially fragmented (Thayer et al., 2013), these changes were not accompanied by any significant alterations in the polarization of the Golgi toward the dendrite, since the number of neurons with zero, one, or two Golgi polarities was not significantly altered in comparison to control neurons (**Figures 5F,G**). These results suggest that enhancing global synaptic activity selectively reduces the net extension of the basolateral dendrite, without significantly affecting the net extension of the apical dendrite.

## Discussion

Regulated dendritic growth forms the basis for wiring of the nervous system. Dendrite asymmetry in pyramidal neurons is critically linked to their functional roles (Xu et al., 2012). Accordingly, various cellular receptors and ion channels are distributed asymmetrically on these dendrites (Kerti et al., 2012; Nusser, 2012) and some behavioral paradigms and molecular effectors differentially affect synapse distribution on apical and basolateral dendrites (Srivastava et al., 2012).

The molecular cues that guide the formation and maintenance of asymmetric dendrite polarity are beginning to be uncovered (De Marco Garcia et al., 2011; Kleindienst et al., 2011; Piatti et al., 2011; Srivastava et al., 2012; Miao et al., 2013; Niisato et al., 2013; Kupferman et al., 2014), however, our knowledge is far from complete. Studies indicate that the specification of the apical and basolateral dendrites is governed by a cell intrinsic program independent of spatially organized extrinsic cues (Horton et al., 2006). These programs are likely critical for neural network wiring. For example, loss of Ube3a in developing neurons leads to alterations in apical basolateral dendrite polarity. Mutations in the gene encoding Ube3a underlie Angelman’s syndrome, a developmental disorder associated with autism and related disabilities (Miao et al., 2013). Thus, programs that regulate dendrite asymmetry are likely critical for appropriate wiring in the developing brain.

It is very likely that molecular mechanisms and neuronal activity cooperate to regulate the generation and maintenance of asymmetry of the dendritic arbor. Our data demonstrate a differential role for cell intrinsic and network neuronal activity in the developmental program that contributes to the asymmetry of the dendritic arbor during development. More importantly, our data indicate that the apical and basolateral dendrites respond differently to cell intrinsic and network activity cues. Interestingly, these alterations in activity do not affect the polarity of the Golgi, suggesting that activity regulates the extension of individual dendrites without affecting their polarization or identity. These results are consistent with a model in which once the identity of the dendrite is specified, each dendrite functions as an autonomous compartment and generates its arbor independently or semi-independently of the other dendrites. Thus, developing dendrites may have dendrite specific control mechanisms for morphogenesis, in addition to global neuronal control mechanisms.

The ability of the different dendrites to respond differentially to activity has important implications for development of the neuronal circuitry. The development of the neuronal circuitry is complex (Bloodgood et al., 2013; Joo et al., 2014). During development, axon guidance and synaptic targeting must be spatially and temporally controlled to ensure correct neural circuit wiring. One mechanism that promotes appropriate wiring is temporal coordination of presynaptic elements and their targets. Temporal control over synapse specificity may occur by restriction of synaptic partner choices. By differentially responding and extending in response to cell intrinsic and neuronal network activity, the dendritic arbor may coordinate neuronal activity with other cues to contribute to the eventual generation of synapse specificity (Bloodgood et al., 2013). Thus, aligning the differential growth of apical and basolateral dendrites to activity cues may provide another mechanism to ensure precise sculpting of neuronal circuit wiring leading to the formation of functional neuronal circuits in the developing hippocampus.

## Author Contributions

Experiments conceived by JA, data collection for all figures, except **Figure 2** – JA, YY, and DS, data in **Figure 2** – ES, data in **Figures 3F,4F**, and **5F** – LY, JA. JA wrote manuscript in consultation with other authors.

## Conflict of Interest Statement

The authors declare that the research was conducted in the absence of any commercial or financial relationships that could be construed as a potential conflict of interest.
